# Mucociliary Clearance Defects in a Murine In Vitro Model of Pneumococcal Airway Infection

**DOI:** 10.1371/journal.pone.0059925

**Published:** 2013-03-19

**Authors:** Manfred Fliegauf, Andreas F.-P. Sonnen, Bernhard Kremer, Philipp Henneke

**Affiliations:** 1 Centre of Chronic Immunodeficiency (CCI), University Medical Centre Freiburg and University of Freiburg, Freiburg, Germany; 2 Department of Paediatrics and Adolescent Medicine, University Medical Centre Freiburg, Freiburg, Germany; Louisiana State University, United States of America

## Abstract

Mucociliary airway clearance is an innate defense mechanism that protects the lung from harmful effects of inhaled pathogens. In order to escape mechanical clearance, airway pathogens including *Streptococcus pneumoniae* (pneumococcus) are thought to inactivate mucociliary clearance by mechanisms such as slowing of ciliary beating and lytic damage of epithelial cells. Pore-forming toxins like pneumolysin, may be instrumental in these processes. In a murine *in vitro* airway infection model using tracheal epithelial cells grown in air-liquid interface cultures, we investigated the functional consequences on the ciliated respiratory epithelium when the first contact with pneumococci is established. High-speed video microscopy and live-cell imaging showed that the apical infection with both wildtype and pneumolysin-deficient pneumococci caused insufficient fluid flow along the epithelial surface and loss of efficient clearance, whereas ciliary beat frequency remained within the normal range. Three-dimensional confocal microscopy demonstrated that pneumococci caused specific morphologic aberrations of two key elements in the F-actin cytoskeleton: the junctional F-actin at the apical cortex of the lateral cell borders and the apical F-actin, localized within the planes of the apical cell sides at the ciliary bases. The lesions affected the columnar shape of the polarized respiratory epithelial cells. In addition, the planar architecture of the entire ciliated respiratory epithelium was irregularly distorted. Our observations indicate that the mechanical supports essential for both effective cilia strokes and stability of the epithelial barrier were weakened. We provide a new model, where - in pneumococcal infection - persistent ciliary beating generates turbulent fluid flow at non-planar distorted epithelial surface areas, which enables pneumococci to resist mechanical cilia-mediated clearance.

## Introduction

Pneumonia can be caused by various pathogens (bacteria, viruses, fungi) and is a leading cause of death due to infectious disease in industrialized countries [1]. *Streptococcus pneumoniae* (pneumococcus) is the major pathogen of community-acquired pneumonia and causes more than one million infant deaths every year worldwide [2]. Pneumococci usually asymptomatically colonize the upper respiratory tract (nasopharynx) of humans [2]. Accordingly, they mainly exist as commensal bacteria along with other co-resident microorganisms [3]. Colonizing pneumococci can persist for weeks in adults or even months in children without any medical sequelae (colonization stage/carrier stage). On occasions, pneumococci pass to other areas where they can cause severe diseases (pathogenic stage). These include the lower airways/lungs (pneumonia), the middle ear (middle ear infections/otitis media), the cerebrospinal fluid of the brain (meningitis), and the blood (bacteriaemia or septicemia), respectively [4], [5]. Although pneumococci are thought to follow similar strategies to attack ciliated respiratory and ciliated ependymal epithelia, the mechanisms that transform the persistently colonizing phenotype to an invasive pneumococcal disease with high morbidity and mortality are poorly understood.

Under normal conditions, the tracheal, bronchial and lung epithelia act as a mechanical barrier and sentinel system against pathogens. The mucus in the tracheo-bronchial tree traps inhaled particles, pathogens and toxins and transports them quickly through the trachea towards the pharynx by means of ciliary beating and cough [6], [7]. Each ciliated epithelial cell carries approximately 200 cilia (∼7–10 µm in length), which move the extracellular mucus by constant, orchestrated and vigorous beating [6], [8].

This “mucociliary clearance” mechanism ensures that inhaled particles do not come into direct contact with the epithelial cells and do not reach the alveolar cavities. Only when pathogens have resisted mucociliary clearance and have spread within the lung tissue, are resident alveolar macrophages required to neutralize the pathogenic bacteria and/or to recruit other elements of innate immunity e.g. via Toll-like receptor-mediated signaling [1], [9]. The importance of the mucociliary escalator is further emphasized by the consequences of its dysfunction. For instance, patients with impaired motile cilia function (PCD, primary ciliary dyskinesia) and patients with highly viscous mucus (as in CF, Cystic Fibrosis) suffer from recurrent and severe sinopulmonary infections that can result in chronic scarring and bronchiectasis [10].

Based on the observations that reduced mucus flow is advantageous for the pathogens to resist airway clearance, it has been suggested that pneumococci (and analogously other airway pathogens) might tightly adhere to ciliated epithelia and/or slow down the ciliary beat. Other possible mechanisms to escape the powerful forces of mechanical clearance include embedding into biofilms, lytic damage or direct invasion of host epithelial cells, or increase of mucus viscosity [11], [12]. However, simplified experimental (murine) models that allow for analysis of the dynamic interaction of pathogenic organisms and the highly specialized ciliated respiratory epithelium were difficult to establish and only recently became available [13], [14].

Pneumolysin is a key virulence factor in pneumococcal infections [2],[5],[15]–[21]. The 53 kDa monomer is released at low concentrations during colonization but at high levels upon bacterial autolysis in later stages of infection. 30–50 monomers assemble a large transmembrane channel (∼260 Å in diameter) that allows for free exchange of ions and small molecules and eventually mediates cell lysis and tissue damage. Purified pneumolysin has been reported to cause rapid, dose-dependent inhibition of the ependymal ciliary beat frequency and simultaneous cell damage. However, a pneumolysin-deficient strain had similar effects when compared to the parental wildtype pneumococcal strain, suggesting additional, pneumolysin-independent disease mechanisms. Other pneumococcal virulence factors include hydrogen peroxide, hyaluronidase and various capsular components [2], [15], [20], [22].

In general, to escape from mucociliary clearance, pneumococci (and other airway pathogens) need to reduce clearance velocity by delaying mucus flow, which in turn allows for bacterial proliferation within the airways. This can be accomplished by a reduction of the ciliary beat frequency as described in several studies [20], [23], [24]–[29]. However, the biological consequences are largely dependent on the complex hydrodynamic properties of the mucociliary clearance mechanisms [8], [30], [31]. For instance, a 30% reduction of the baseline beat frequency - 12 versus 15 beats per second - is still within the normal range of healthy individuals [32]–[34] and alone might not severely impair mucus flow. Pathogenic mechanisms that could cause inefficient mucociliary clearance without ciliary beat slowing, increasing mucus viscosity or epithelial cell damage have not been reported yet.

In this study we investigated how pneumococci resist mucociliary clearance by monitoring the function of the ciliated epithelium during pneumococcal infections. We employed a murine *in vitro* model, to determine the ciliary beat frequency by high-speed video microscopy and to evaluate the epithelial fluid flow velocity by time-lapse live-cell imaging. The morphological changes in the infected respiratory epithelium were analyzed by four-color confocal fluorescence microscopy and were confirmed *ex vivo*. We found that within the first 3–4 hours after inoculation, pneumococci do not alter the ciliary beat frequency. Instead they cause severe lesions in the F-actin cytoskeleton that affect both the architecture and the mechanical stability of the ciliated respiratory epithelium. Our results indicate that in pneumococcal airway infection, persistent ciliary beating together with an aberrant epithelial geometry impairs the hydrodynamics of mucociliary clearance.

## Materials and Methods

### Ethics Statement

All animal experiments were performed in compliance with the German animal protection law (TierSchG). The mice were housed and handled in accordance with good animal practice as defined by FELASA (www.felasa.eu/guidelines.php) and the national animal welfare body GV-SOLAS (www.gv-solas.de/index.html). The animal welfare committees of the universities of Freiburg as well as the local authorities (Regierungspräsidium Freiburg) approved all animal experiments.

### Air-liquid Interface Cultures of Murine Ciliated Respiratory Epithelia

Ciliated respiratory epithelia were cultured as described previously with minor modifications [13], [14]. Briefly, epithelial cells were enzymatically isolated from dissected mouse trachea with Dispase II (Roche, Mannheim, Germany) and fibroblasts were removed by adherence. Epithelial cells were cultured until confluency was reached (6–8 days; 3–4 medium changes) in phenolred-free DMEM/F12 (Invitrogen, Karlsruhe, Germany) supplemented with 5% Ultroser G (Pall, Dreieich, Germany) and 5% antibiotic-antimycotic (Invitrogen) on Transwell cell culture inserts (Polyester, 0.4 µm pore size, 12 mm diameter; Corning-Costar, Amsterdam, The Netherlands) coated with Type IV collagen (Sigma, Taufkirchen, Germany). Ciliogenesis was then induced with DMEM/F12 supplemented with 5% NU-serum, 0.1% ITS-premix (both from BD Biosciences, Heidelberg, Germany) and 100 nM all-trans retinoic acid (ATRA; Sigma) and by exposure of the apical cell surface to air. Apical surfaces were rinsed with every medium change (3 times per week). Cultures were maintained for up to 6 weeks and developed ciliated epithelial structures of variable size in the central area with high percentage (50±30%) of ciliated cells and vigorous ciliary beating. The versatility of the respiratory epithelial cultures was limited by their heterogeneity, which was dependent on the amount of co-cultured non-epithelial cells in the peripheral areas. For *in vitro* infections, air-liquid interface cultures were washed 5–6 times with phosphate buffered saline (PBS) to remove antibiotics. Medium at the basal side was replaced with medium without antibiotics and bacteria were added in a total volume of 150 µl to the apical epithelial side.

### Culture of Pneumococci and Preparation of an Inoculum

The parental wildtype strain of *Streptococcus pneumoniae* D39 (serotype 2) which is highly virulent in mice [5], [35]–[38] and its pneumolysin-deficient derivate, plyA(−), were grown in a 1∶1 mixture of DMEM (Biochrom, Berlin, Germany) and FBS (Lonza, Basel Switzerland) at 37°C and 5% CO_2_ without agitation to an OD_600_ of approximately 0.2 which corresponds to 1×10^8^ bacteria per ml. For *in vitro* infections, bacteria were diluted 1∶2–1∶4 in 150 µl total volume of 50%DMEM/50%FBS and added directly to the apical cell side of respiratory epithelial cultures. For *ex vivo* infections, diluted bacteria were directly added to tracheal tissue explants. When not stated otherwise, viable pneumococci were used in early log phase directly from growing cultures. Bacteria growth was continuous during the infection intervals. To avoid bacterial overgrowth in prolonged infection experiments, bacteria were diluted 1∶2 during the infection. Infection samples achieved a final OD_600_ of 0.2 to 0.35 and bacterial viability was confirmed by plating aliquots on blood agar plates. Other bacterial species that were used as controls – either pathogenic controls (including *Haemophilus influenzae*, Group-B streptococcus, *Staphylococcus aureus*) or “metabolic active” non-pathogenic controls (laboratory strains of *E.coli*) - were grown and prepared for inoculation following analogous protocols. Bacterial growth characteristics were not equalized. Group B streptococci, *E. coli* and *S. aureus* usually had much faster growth kinetics and reached much higher densities, in contrast to pneumococci.

### Ex vivo Airway Infection Model

Tracheae were immediately dissected from sacrificed mice and pharynx, bronchi, residual connective tissue, fat and muscles were removed using a dissecting microscope (Zeiss), leaving the outer cartilage wall and the inner epithelium intact. Tracheae were opened lengthwise and cut into two halves when applicable. Tissue pieces were transferred to appropriate cell culture vessels (e.g. 24-well plate sized) and submerged in 500 µl medium (50%DMEM/50% FBS). 500 µl of a pre-diluted pneumococcus culture were added for *ex vivo* infection experiments to achieve starting CFUs of 1×10^5^–1×10^6^.

### Determination of the Ciliary Beat Frequency by High-speed Video Analysis

Ciliary beat frequencies were determined with the SAVA system (Sisson Ammons Video Analysis of ciliary beat frequency [39]. Air-liquid interface cultures were viewed at room temperature with an Zeiss Telaval31 phase contrast microscope (10x, 20x and 40x objectives; Carl Zeiss, Jena, Germany) equipped with a Basler monochrome high-speed video camera (Ahrensburg, Germany) set at 100 or 125 frames per second (2 or 2.6 seconds recordings; 640×480 pixel). Due to the inherent optical properties of the porous membrane and the transwell system, resolution was limited and only evaluation of the ciliary beat frequency, but not of the beat pattern and beat coordination, was accurate. The SAVA software also calculates the “active area” which we used as a surrogate marker for the percentage of cells carrying actively beating cilia per analyzed visual field (typically 50±30% in the central area of air-liquid interface cultures). To avoid experimental bias, beat frequencies were only evaluated when active areas were >20%.

### Determination of the Epithelial Fluid Flow Velocity

Cilia-driven extracellular fluid flow was visualized by adding 3 µm blue dye-filled beads (Polybead Microspheres; Polysciences, Eppelheim, Germany) to the apical epithelial side of air-liquid interface cultures. Beads were blocked with FBS and diluted in PBS prior to usage. Transport of the beads along the surface was recorded on a Zeiss Axiovert 200 M, with a 40x phase contrast objective using the AxioVision software (Zeiss). Tracking of individual beads and calculation of the transport velocity was done with a multi tracking tool in ImageJ [40] on time-lapse recordings (video length 60 sec; 0.5 frames per second). During video recordings, *in vitro* infection samples were removed from the incubator (37°C; 5% CO_2_) and kept at room temperature. Infection intervals were therefore increased to 4 h. Analogous bead velocity calculations from SAVA recordings were non-accurate due to the short video duration. Simultaneous determination of the ciliary beat frequencies during time-lapse video microscopy was not possible because the two separate microscope setups could not be combined.

### Immunofluorescence Staining and Confocal Imaging

For morphological analyses after treatment, transwell cell culture inserts with the epithelial cultures attached, or tracheal tissue explants, respectively, were washed with PBS and submerged in 4% paraformaldehyde for fixation. Membranes were removed from the plastic support and cut into halves. Samples were permeabilized for 10 minutes with, 0.2% Triton-X 100 and blocked for 1 hour with 0.5% skim milk in PBS-T (PBS plus 0.05% Tween-20). Incubations with primary (at least 1 hour) and secondary (1 hour) antibodies were carried out at room temperature. The following antibodies were used: mouse-anti-acetylated-α-tubulin (Sigma) as a cilia marker; rabbit-anti-pneumococcus serum (Statens Serum Institut, Copenhagen, Denmark); goat-anti-mouse Alexa Fluor488 and goat-anti-rabbit Alexa Fluor546 secondary antibodies (Molecular Probes, Invitrogen). After immunolabelling, DNA and F-actin were simultaneously stained with Hoechst33342 (Sigma) and Phalloidin-Atto633 (Atto-Tec, Siegen, Germany). Samples were repeatedly washed with PBS-T after each step. Confocal-3D images (Z-stacks) were taken on a Zeiss laser scanning microscope LSM710, with an Axiovert 200 M equipped with 63x and 100x oil immersion objectives (Carl Zeiss, Jena, Germany). Four-color fluorescence images were recorded in the channel mode, with 512×512 or 1024×1024 pixels, in line scan mode with two- or four-fold average sampling settings. Evaluations and 3D reconstructions were done with the Zeiss ZEN2009 or ZEN-black software.

## Results

### In vitro Pneumococcal Airway Infection does not Affect the Ciliary Beat Frequency in Respiratory Epithelia

Airway pathogens must have evolved strategies that allow them to resist cilia-mediated clearance e.g. via inhibition of ciliary motility. We argued that a mild overall reduction of the beat frequency - e.g. during initiation of airway disease by a limited number of pathogens - might be insufficient to inactivate the mucociliary clearance mechanism due to its complex hydrodynamic properties. We therefore investigated the functional consequences for the ciliated respiratory epithelium when contact with pathogenic *Streptococcus pneumoniae* is established. We used an air-liquid interface culture system of mouse tracheal epithelium for modeling pneumococcal airway infections *in vitro*. Terminally-differentiated respiratory epithelia which are characterized by vigorous ciliary beating were infected by application of bacteria to the apical (ciliated) cell side. In addition to wildtype pneumococci (strain D39), its isogenic pneumolysin-deficient variant plyA(−) as well as purified recombinant pneumolysin was used to determine the role of the pneumococcal key virulence factor.

**Figure 1:**
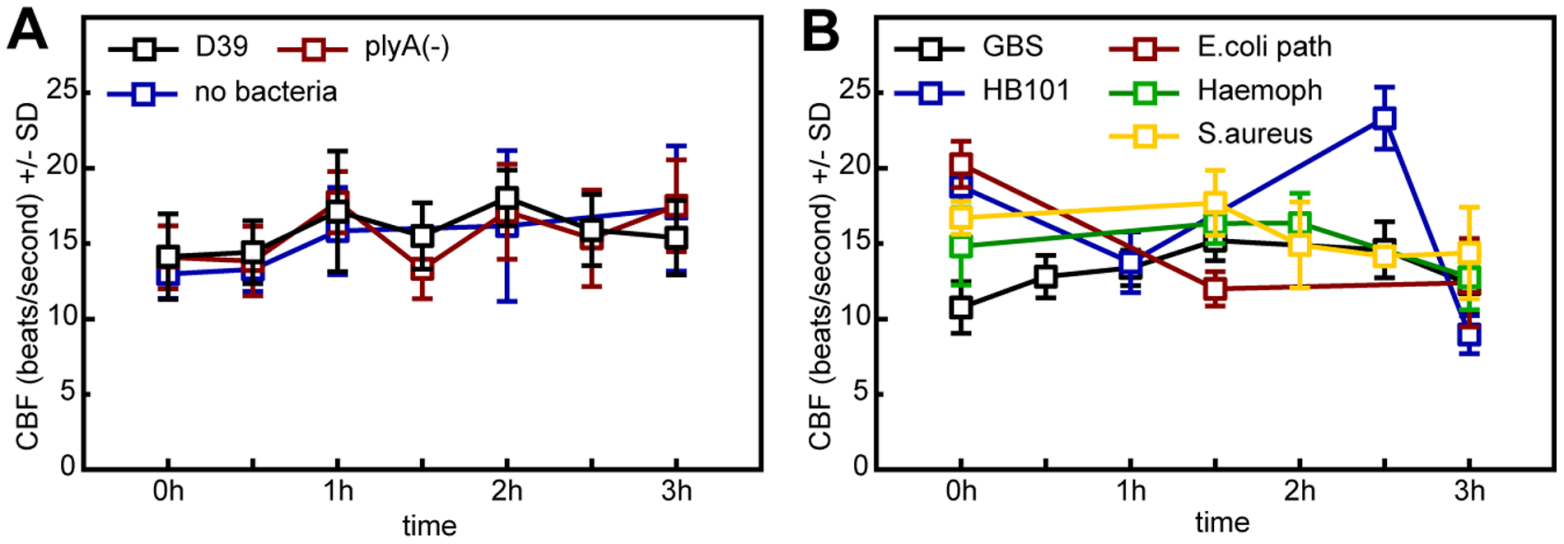
Pneumococci do not affect the ciliary beat frequency. In ciliated respiratory epithelial cells derived from mouse trachea that are induced to undergo ciliogenesis in air-liquid interface cultures, a typical ciliary beat frequency of 15±5 Hz (beats per second) was regularly observed. Slight random and temporary variations in beat frequencies occurred upon handling of the cultures and sporadically exceeded 25 Hz. In each individual experiment, the apical (ciliated) epithelial side of one culture was infected with viable bacteria and beat frequencies were measured in various epithelial areas at different time points. Each box indicates the mean beat frequency within a ∼30 min interval as indicated. Bars depict the standard derivation (SD) of results from individual experiments. Lines connect the results for each bacterial species or control, respectively. (**A**) Persistent beating (15±5 Hz) was observed upon *in vitro* infection with pneumococci (D39, wildtype strain; plyA(−), pneumolysin-deficient derivate) within 3 hours after addition of bacteria as in controls. D39∶199 measurements in 8 independent experiments; plyA(−) 66/3; no bacteria: 74/3 (**B**) Robust maintenance of the baseline ciliary beat frequency within 3 hours after infection with pathogenic and non-pathogenic *Escherichia coli* (HB101) as well as with the airway pathogens Group-B streptococci (GBS; NEM316), *Haemophilus influenza* and *Staphylococcus aureus*. GBS: 70 measurements in 2 independent experiments; *E.coli* path: 30/2; HB101∶28/1; *Haemoph*: 35/2; *S.aureus*: 41/2.

We found that in heavily ciliated areas (i.e. 50±30% ciliated cells), which closely resemble respiratory epithelia *in vivo*, ciliary beat remained at remarkably robust frequencies (Figure 1) within a small range (15±5 beats per second). Beat frequency only varied temporarily, depending on alteration of conditions or treatments such as temperature changes during microscopic analyses or mechanical stimulation by addition of fluid and particles (data not shown). Surprisingly, apical infection with viable pneumococci (5×10^6^–1×10^7^ CFU/ml; MOI ∼10; with up to 4 h inoculation) did not affect the ciliary beat frequency *per se* (Figure 1A), which remained within the normal range (15±5 beats per second). Accordingly, under these conditions, the ciliary clearance mechanism was maintained and bacteria were moved off the ciliated areas and did not actively or tightly adhere to the epithelial surface. Persistent ciliary beating at normal frequencies was also observed in MOCK-infected controls and pneumolysin-deficient pneumococci (Figure 1A) as well as with other lung-pathogenic and non-pathogenic bacterial species (Figure 1B). Furthermore, no effects were detectable when heat-, ethanol- and antibiotics-inactivated pneumocooci were used, or upon treatment with recombinant pneumolysin (up to a very high concentration of 10 µg/ml; data not shown).

In summary, in intact ciliated respiratory epithelia grown *in vitro*, beat frequency was maintained within the first hours after apical infection with pneumococci. Reduced ciliary beating was not observed, neither in pneumococci- nor in control infected samples, unless overall epithelial damage became clearly obvious due to bacterial overgrowth and consequent non-specific toxic effects (e.g. depletion of nutrients or acidification) during prolonged infections (data not shown).

### Pneumococci Require Normal Beat Frequencies to Cause Inefficient Respiratory Epithelial Fluid Flow

**Figure 2:**
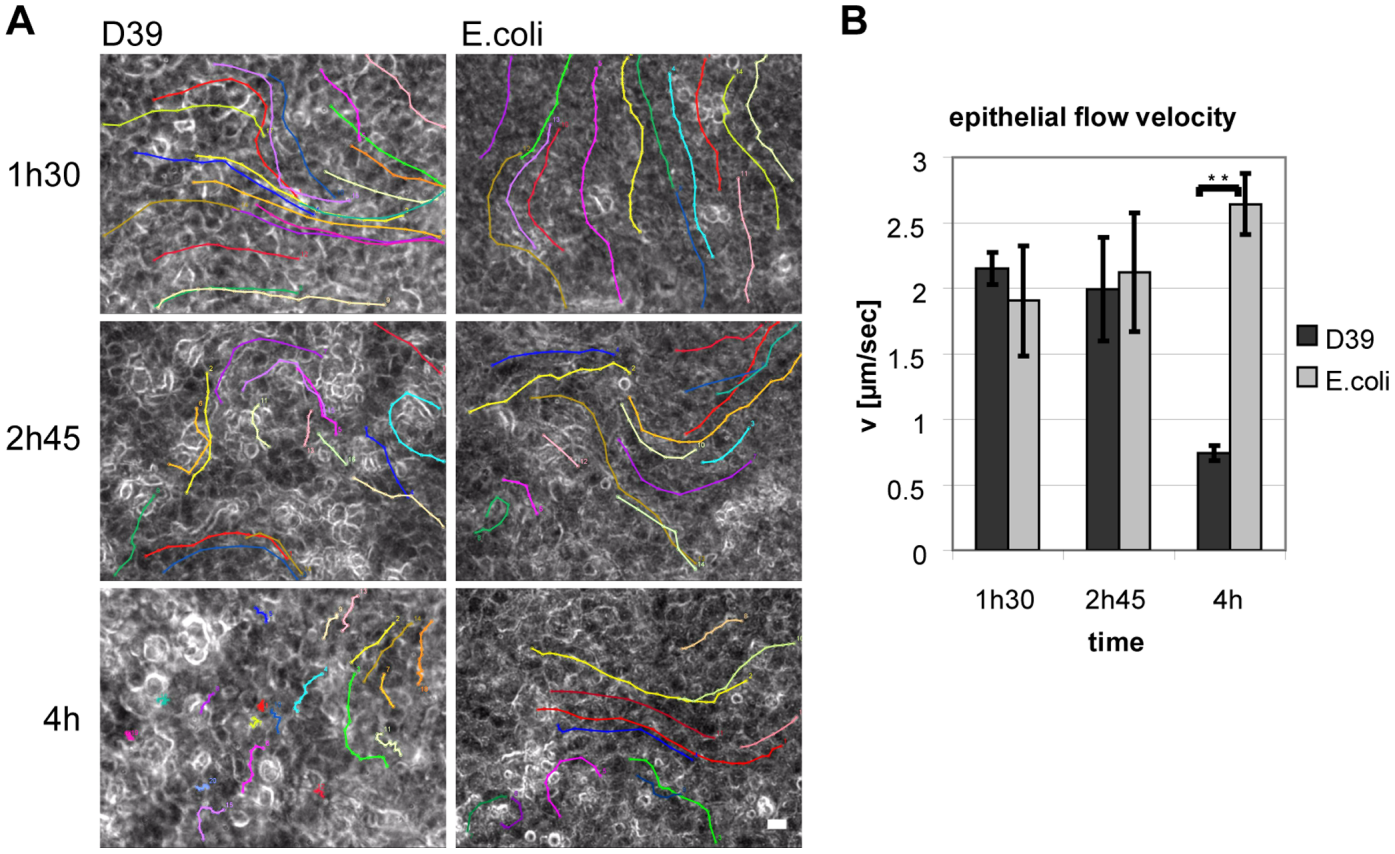
Pneumococci decrease epithelial surface fluid flow and particle transport. Cilia-dependent epithelial surface fluid flow was visualized by time-lapse video microscopy (0.5 frames per second; total video length 60 sec) after addition of dye-filled beads to the infected samples. Videos were recorded at room temperature and the infection interval was increased to 4 h, accordingly. Colored tracking lines were annotated in the last image of each analyzed video and represent transport directions and traveling speed of individual beads. Only areas with vigorous ciliary beating were analyzed. (**A**) Within ∼3 h after apical infection with pneumococci (D39), epithelial particle transport and consequently, fluid flow velocity remained at baseline levels and was undistinguishable from controls. By 4 hours, particle transport speed was severely reduced in the pneumococcus-infected samples and transport directions were partially random. White areas on pneumococci infected epithelia suggest emerging morphological aberrations. Scale bar: 10 µm (**B**) Tracking of at least 60 beads and 4 videos per time point were assessed to calculate the cilia-dependent epithelial flow velocity, which was significantly reduced 4 h after pneumococcal infection (0.74±0.06 µm/sec) compared to controls (2.64±0.23 µm/sec). Statistical analysis was performed by t-test. Error bars represent SEM.

Efficient surface particle transport can only occur when a laminar epithelial fluid flow is established by coordinated and effective ciliary beating [41]. Upon apical infection with pneumococci, evaluation of high-speed video recordings regularly indicated that ciliary beat – although at normal frequencies – was weak and less efficient (Supplementary Video S1). However the loss of “beat efficiency” was also not caused by loss or damage of cilia *per se* (see morphological analyses below). To visualize and quantify the ciliary clearance efficiency we added dye-filled beads (3 µm) to the apical side of the epithelium during pneumococcal infection and determined the velocity of fluid flow-dependent particle transport along the epithelial surface by time-lapse video microscopy (Figure 2). Tracking of individual beads revealed that pneumococci impaired ciliary clearance in a time-dependent manner. This was indicated by severely disturbed direction and velocity of particle transport as compared to infections with non-pathogenic *E.coli* that were used as “metabolic active controls”. The increased appearance of irregular surface structures during pneumococcal infection led us to test the hypothesis whether pneumococci cause morphological alterations of the epithelial architecture that interfere with the conversion of vigorous cilia-beating into proper laminar fluid flow at the surface.

### Pneumococci Cause Loss of the Planar Epithelial Architecture through Targeting of F-actin Cytoskeleton in Ciliated Respiratory Epithelial Cells

We next used three-dimensional (3D) confocal fluorescence imaging to test whether morphological aberrations of the ciliated respiratory epithelia grown *in vitro* accounted for the observed reduced clearance efficiency upon contact with pneumococci. Air-liquid interface cultures were prepared to simultaneously visualize nuclei (Hoechst staining), cilia (with antibodies for the cilia-specific acetylated α-tubulin isoform) and the F-actin cytoskeleton (phalloidin staining), respectively. The role of the F-actin cytoskeleton in ciliogenesis and epithelial differentiation has been well described [42]–[44].

**Figure 3:**
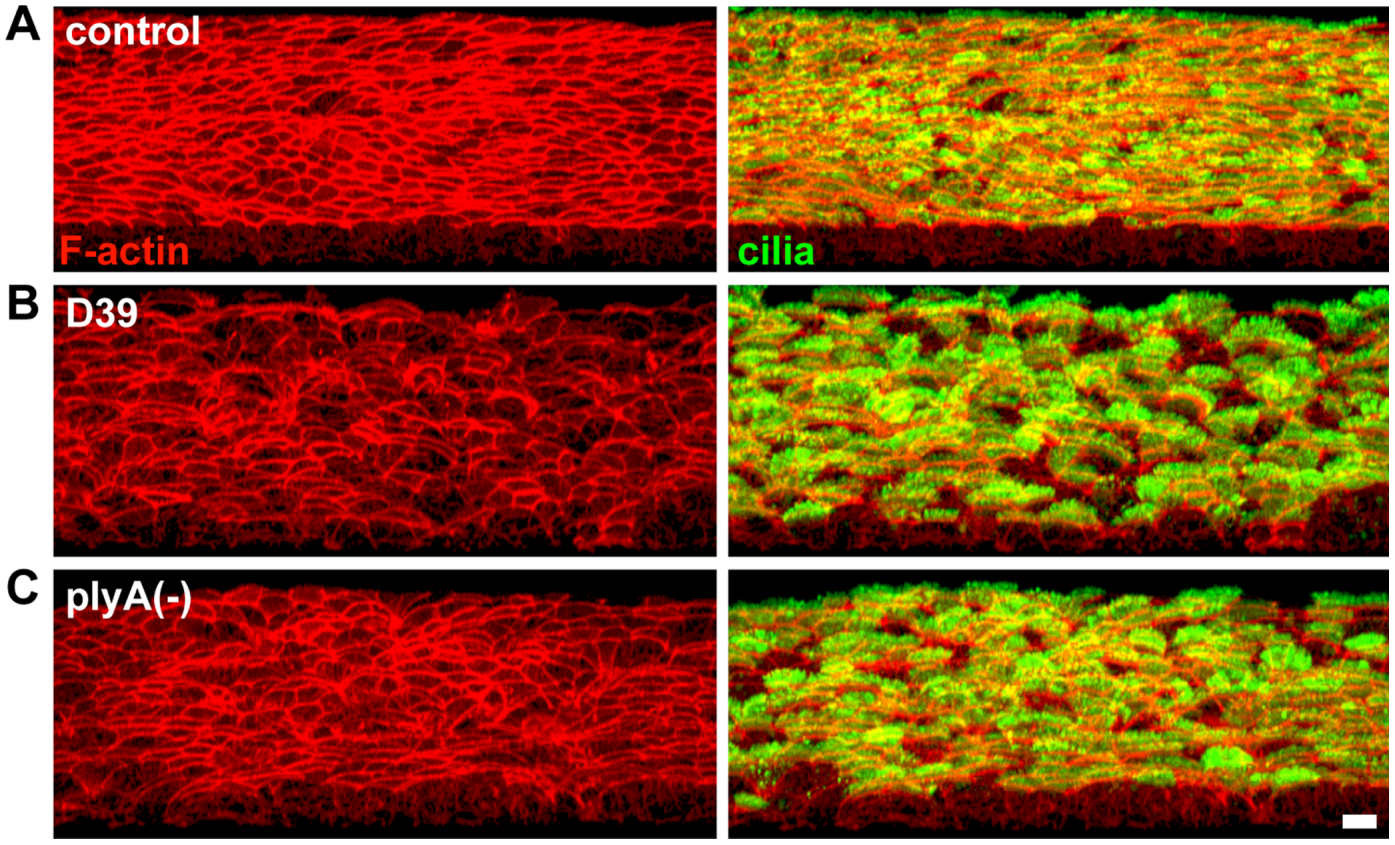
Pneumolysin-independent disruption of the F-actin cytoskeleton causes loss of planar epithelial surface architecture. Heavily ciliated respiratory epithelia were infected (3 h) *in vitro*. 3D profile views of the F-actin cytoskeleton alone (phalloidin staining, left panel) and with cilia (acetylated α-tubulin, right panel) were assembled from confocal Z-stack images. Bacteria, which were only found sporadically attached to the ciliated surface after sample processing and nuclei are not shown. Scale bar: 10 µm. (**A**) The normal planar epithelial morphology is built on a stable “honeycomb-like” F-actin cytoskeleton. It is composed of abundant F-actin at the junctional and apical cell cortex, and is maintained after control inoculation with non-invasive *E.coli*. (**B**) Upon pneumococcal infection (D39, parental wildtype strain), the F-actin cytoskeleton is converted into a non-stable “net-like” structure. Reduced apical F-actin indicates the loss of the mechanical support for efficient cilia strokes. The cell junctions remain closed, although severe distortions of the junctional F-actin occur. The non-planar alignment of aberrantly deformed, dome-shaped cells indicates that persistent ciliary beating generates fluid flow turbulence. (**C**) The comparable severity of epithelial damage after infection with pneumolysin-deficient, plyA(−), and -sufficient pneumococci, suggests a pneumolysin-independent mechanism.

In a pseudostratified ciliated respiratory epithelium, with columnar shaped epithelial cells and a planar surface, the F-actin cytoskeleton constituted a characteristic “honeycomb-like” architecture, composed of two morphological and functional distinct elements (Figure 3A, S1 A–D left panel, S2 A–C, S3 A,C). The predominant F-actin staining was detected at the apical cortex of the cell bodies, at the tightened lateral cell junctions, (“junctional F-actin” [43]). The junctional F-actin, which co-localized with the tight junction protein ZO-1 (zona occludens-1, Figure S2 A–C) is not only essential for maintenance of the columnar cell shape, the basal-to-apical polarity and the epithelial tight junctions, but also for the rigidity and mechanical stability of the epithelium [45]. To a much lesser extend, F-actin was also apparent at the lateral cell borders, probably supporting these functions. The second key component of the F-actin cytoskeleton localized to the planes of the apical cell sides, beneath the cytoplasmic membrane (“apical F-actin” [43] (Figure 3A, S1 A–D left panel, S2 A–C, S3 C). It frames each single ciliary base, and thus anchors the motile cilia at the apical cell side [42]. Moreover, it probably provides the rigidity of the proximal cell aspect and the mechanical support for effective and powerful cilia strokes, both of which are essential for efficient cilia-dependent clearance.

During the first hours after infection with pneumococci, no cilia-loss, cilia shortening or other damage to the cilia was detectable. In contrast, we observed characteristic and progressive lesions in the F-actin cytoskeleton that affected both the cellular and epithelial architecture (Figure 3 B–C, S1, S3 A–B). Pneumococci-infected epithelia appeared with an irregular and rough distortion of the apical ciliated surface. The stable (“honeycomb”) architecture of the F-actin cytoskeleton was aberrantly converted into a weak, unstable “net-like” structure in which the columnar shape of the cells and their planar alignment was lost. The cells remained in close contact and frequent opening of the tight junctions was not observed, in contrast to infection with Group B Streptococci (Figure S1 E–I left panel, S2 D). These overall structural aberrations suggest that persistent ciliary beating (Supplementary Video S2) can no longer maintain a laminar epithelial fluid flow. Instead, it generates turbulence due to the non-planar epithelial surface. This results in inefficient clearing.

### Pneumococci Attack Two Key Elements of the F-actin Cytoskeleton in Ciliated Respiratory Epithelial Cells

**Figure 4:**
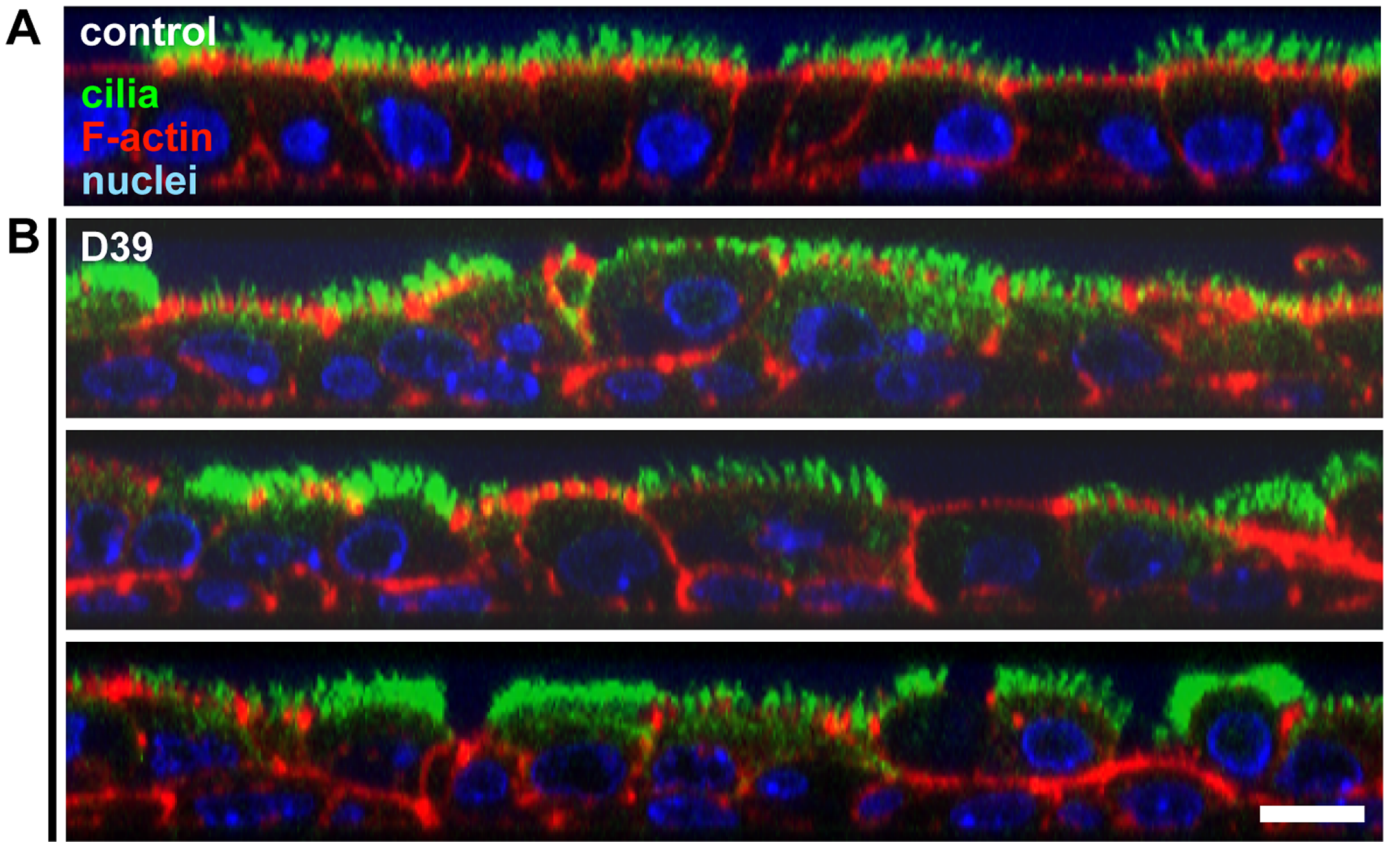
Pneumococci cause disintegration of the ciliated epithelium by F-actin reorganization and partial undocking of cilia. Respiratory epithelia were analyzed for morphological changes after infection by staining of cilia (green), F-actin (red) and nuclei (blue) with confocal Z-stack imaging (orthogonal views). Scale bar: 10 µm. (**A**) The respiratory epithelium is characterized by a strictly planar alignment of columnar-shaped cells, exclusive apical localization of cilia and basal positioning of the nuclei. Prominent F-actin is detected at the junctional and apical cell cortex (where the ciliary bases are anchored) and to a much lesser extent at the lateral cell borders. (**B**) Infection with pneumococci (D39, 2 h45 min) causes a disorganization of the polarized, planar epithelial structure, including the cell shapes and all analyzed sub-cellular compartments. The cytoplasmic space is frequently reduced and the basal positioning of nuclei is lost. Mislocalization of F-actin to the lateral cell borders occurs and a high proportion of cilia detach from the apical cell side, probably due to damage to the apical F-actin (Figure S3). Hence, persistent ciliary beating might only generate insufficient extracellular fluid flow.

Detailed characterization of the F-actin alterations that coincided with the overall structural changes in the respiratory epithelium upon pneumococcal infection revealed a massive mis-localization of F-actin along the entire length of the lateral cell borders (Figure 4, S1 C–D). A large proportion of cells was shifted in the baso-apical direction (up- or downwards) and had been forced into a dome-like shape. Consequently, some cilia of the affected cells were laterally displaced and thus no longer contributed to the planar brush border architecture (Figure 4). These aberrations of the junctional F-actin suggest that persistent beating of laterally-displaced cilia can only produce turbulent instead of laminar epithelial flow. We also frequently observed a reduction in the height of the entire epithelium, obviously caused by a partial collapse of the columnar cell shape (Figure 4) which most likely indicates not only the loss of mechanical stability but also a loss of the strict epithelial polarity.

Progressive destruction of the apical F-actin was indicated by reduced or even absent phalloidin staining at the apical cell cortex (Figure 3, 4, S1 A, S3 C–D). In such cells, staining of monomeric G-actin was increased, suggesting either depolymerization of F-actin or a disturbed G-actin nucleation (data not shown). Thus, the cytoskeletal F-actin elements, which surround the cilia at their bases and probably anchor the cilia (their basal bodies) to the cell body, were affected by pneumococcal infection. Accordingly, cilia were frequently found detached from the apical cell side (Figure 4, S3 E–F), presumably due to loss of the mechanical F-actin support and basal body undocking. The consequence would be inefficient ciliary beating at low amplitudes.

Damage to a similar extent was also observed with pneumolysin-deficient pneumococci (Figure 3, S3), indicating a pneumolysin-independent mechanism. However no effects were observed when inactivated pneumococci (by treatment with heat, ethanol or antibiotics) or supernatants from pneumococcal cultures in early log phase growth were used (data not shown). This observation suggests that metabolic activity of pneumococci is essential. We were unable to obtain firm evidence for direct contact between pneumococci and respiratory epithelial cells being required to attack the ciliated epithelium, as only sporadic bacteria remained attached to the epithelial surface after immunofluorescence staining and we could not assess whether they adhered actively or passively (data not shown). Prolonged infection and/or pneumococcal overgrowth caused progressive lesions with almost completely irregular epithelial structures (Figure 4, S1 A–D), indicating that the columnar cell shape could no longer be maintained. In addition, destruction of the epithelial barrier, and detachment of the cells from the membrane support occurred (data not shown).

### Ex vivo Pneumococcal Airway Infection of Tracheal Explants Cause Severe Epithelial Lesions that Inactivate Mucociliary Clearance Function

**Figure 5:**
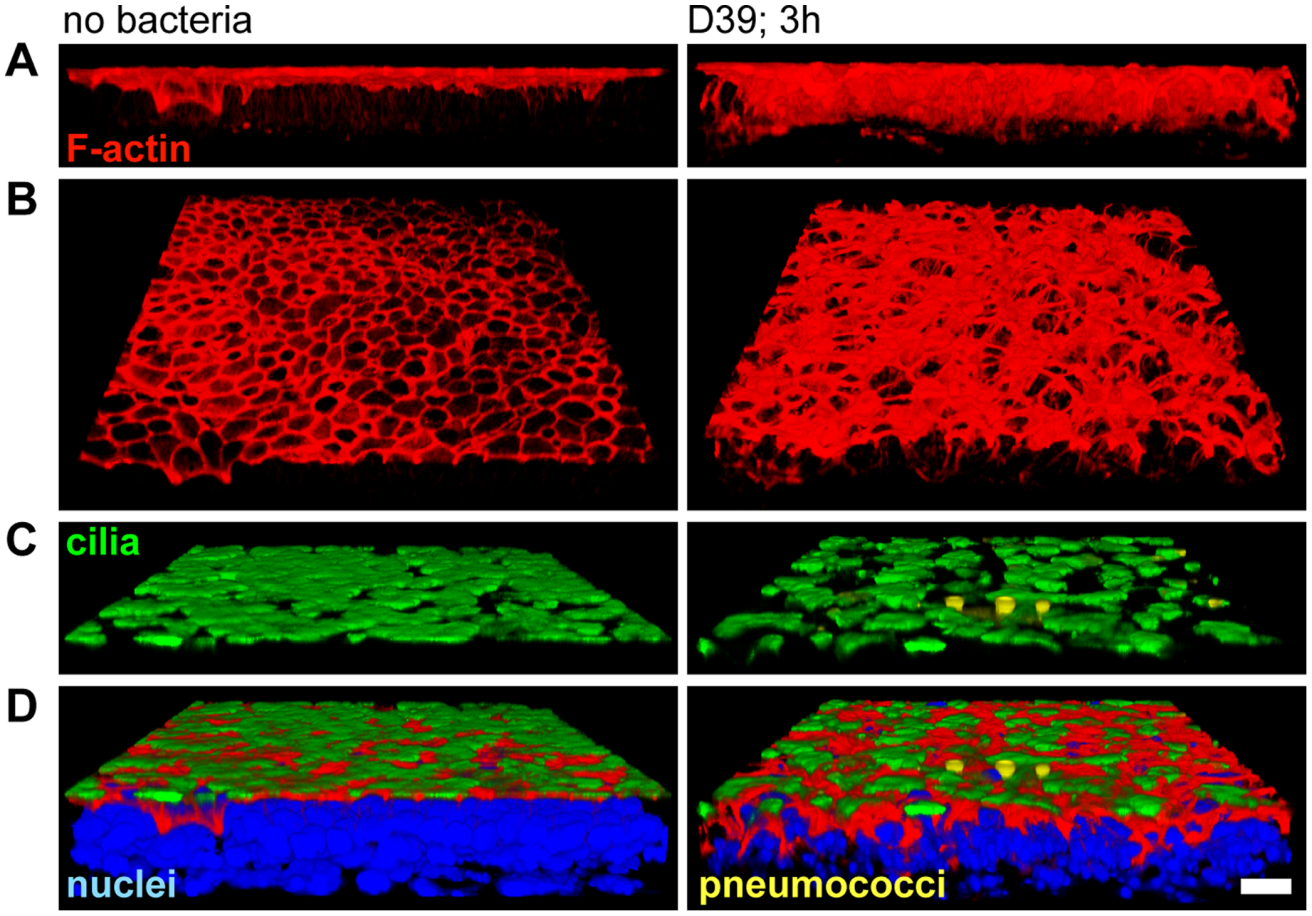
*Ex vivo* pneumococcal airway infection causes distortion of the F-actin cytoskeleton and of the planar epithelial architecture. Freshly dissected mouse tracheae were untreated (left) or infected with pneumococci (3 h; right) and analyzed for morphological changes by confocal fluorescence microscopy. 3D illustrations were assembled from z-stack images. Scale bar: 10 µm. (**A**) The normal morphology of the F-actin cytoskeleton in ciliated tracheal epithelium is characterized by a robust F-actin cytoskeleton. In 3D side views, abundant F-actin staining is only observed at the apical cell sides (untreated control, left). In pneumococcal infected samples, massive mis-localization of F-actin throughout the entire cell body area occurs (right). (**B**) 3D profile views show that upon infection with pneumococci the stable F-actin cytoskeleton of the tracheal epithelium (left) is converted into an irregular structure (right). (**C**) The planar ciliated epithelial surface of the tracheal epithelium (left) is irregularly distorted in the infected sample (right). Single pneumococci sporadically adhered to the ciliated surface (3D profile view). (**D**) Overlay images, showing the collapse of the polarized, planar organization of the ciliated tracheal epithelium after pneumococcal infection (3D profile view).

Our findings indicated that the ability of the pneumococcus to resist mucociliary clearance might be based on specific aberrations of the respiratory tissue morphology. *In vitro*, the basal-to-apical polarity is well established through the air-liquid interface. However, the model lacks a “distal-to-proximal” polarity (analogous to the lung-to-pharynx polarity of the lower airways) and thus the lateral alignment of the cells, as well as the direction of ciliary beating and fluid flow, is partially random. To validate our findings with isolated airway tissue in an *ex vivo* infection model (Figure 5), we prepared tracheal explants preserving the ciliated epithelium. Confocal imaging showed that the morphological and functional complexity of the ciliated tracheal epithelium is not reproduced *in vitro*. The tracheal epithelium that lines the cartilage tube (composed of flexible ring-like structures) is morphologically discontinuous. The ring-like proportions of the ciliated epithelium are separated by a row-like alignment of less ciliated epithelial structures that probably span the flexible cartilage parts (Figure S4 A–B).

Upon *ex vivo* infection with pneumococci we found that the F-actin cytoskeleton was affected to a similar extent as observed *in vitro* (Figure 5, S4). F-actin staining was no longer detected predominantly at the apical cell side, but was highly accumulated in the baso-lateral aspects. In addition, the columnar cell shape, the planar alignment of the ciliary brush border and the planar epithelial surface were distorted. Increased adherence of pneumococci was not observed. Therefore, our results from both pneumococcal airway infection models are consistent.

**Figure 6:**
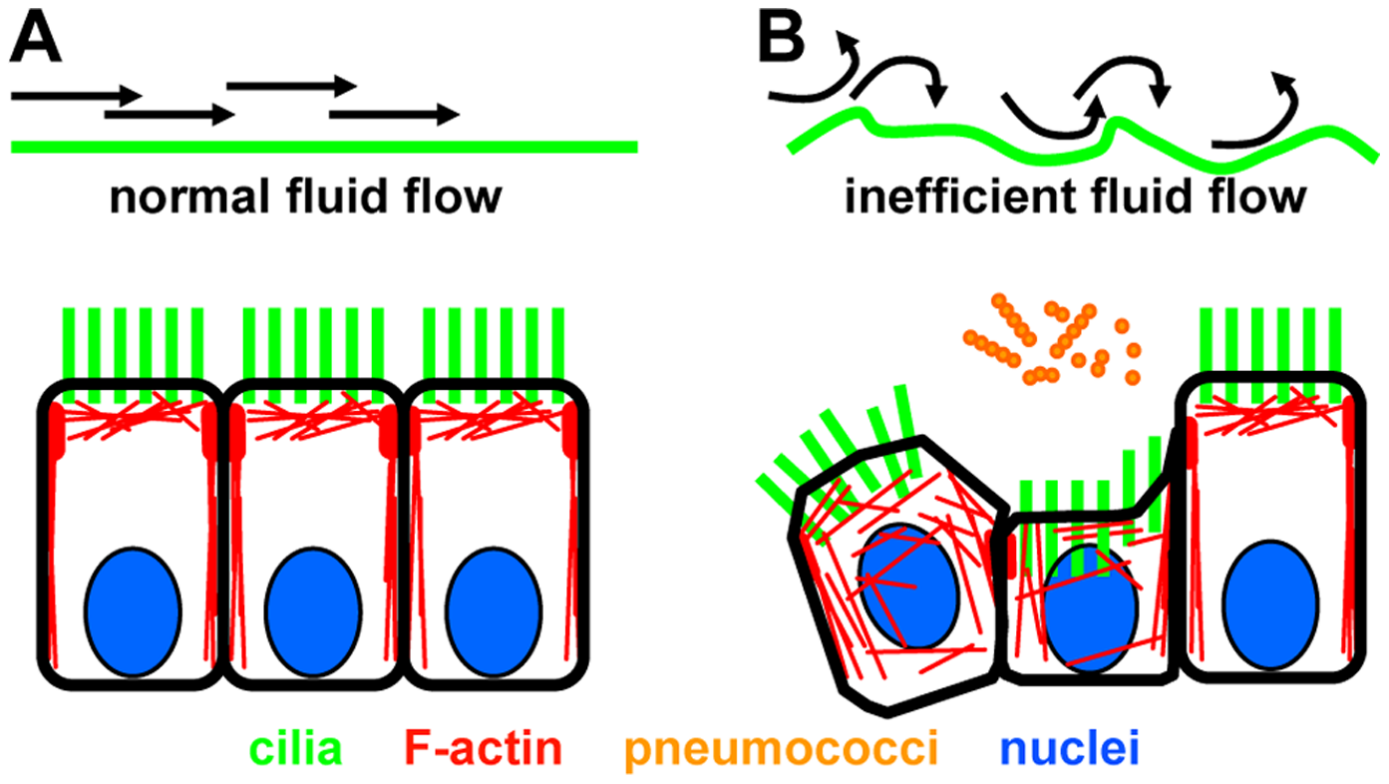
Possible mechanism which could enable pneumococci to resist mucociliary clearance. (**A**) Columnar shaped epithelial cells generate powerful cilia strokes and efficient extracellular flow along the planar, mechanically stable surface. (**B**) Pneumococcal infection causes F-actin mislocalization, disintegration of the epithelial architecture, weakened mechanical stability, detachment of cilia and powerless cilia strokes. Persistent ciliary beating generates local turbulence. Impaired clearance is independent of reduction in ciliary beat frequency.

Our observations propose a concept in which pneumococci resist cilia-dependent mechanical airway clearance by interfering with the planar epithelial architecture leading to non-laminar, turbulent epithelial flow (Figure 6). Here, the “inefficiency” of the ciliary beat (at normal frequencies) is secondary to the loss of mechanical properties of the ciliated epithelium but is independent of lytic cell damage or slowing of cilia motility.

## Discussion

We used both *in vitro* and *ex vivo* airway infection models, to analyze the functional and morphological consequences of the ciliated respiratory epithelium when contact with pneumococci is established. Under healthy conditions, inhaled pathogens are trapped in airway mucus and are transported out of the airways by means of mucociliary clearance. The clearance efficiency is dependent on several prerequisites including an effective ciliary beating pattern, a beat frequency of 12–15 Hz, synchronized coordination of all cilia in the epithelium (metachronal wave) and – as our results confirm – a mechanically robust architecture with planar epithelial alignment of polarized columnar-shaped ciliated cells. In order to colonize the respiratory tissue, airway/lung pathogens such as the pneumococcus need to inactivate the mucociliary clearance mechanisms. This might be accomplished by several strategies (or their combination), including slowing of the ciliary beat and increasing mucus viscosity. Both cause reduced mucus transport which then allows the pathogens to come into close contact with the epithelial surface. Firm adhesion to the respiratory cells is followed by bacterial proliferation, lytic damage of the epithelium and invasion. We found that although particle transport was markedly reduced the ciliary beat frequency remained within the normal range (Figure 1–2). Instead, pneumococci damaged the F-actin cytoskeleton in a way that affected the polarized columnar shape of the individual ciliated respiratory epithelial cells and their planar alignment (Figure 3–5). Our results propose a mechanism in which pneumococci resist cilia-dependent mechanical airway clearance by interfering with the mechanical properties and the planar architecture of the respiratory epithelium (Figure 6). As a consequence, persistent ciliary beating generates turbulent - instead of laminar - epithelial flow that is unable to transport particles and bacteria along the surface.

In such a hydrodynamic mechanism, spatially restricted effects would be sufficient to resist clearance. Theoretically, a limited number of pneumococci could cause local turbulence maintained by persistent ciliary beating, which then generates “niches” in which the bacteria can proliferate and escape from clearance. Thus, tight physical interaction between pneumococci and respiratory epithelial cells would not be required. In support of this view, the first detectable lesions occurred in a time-dependent, dynamic process, at restricted surface spots, i.e. not simultaneously on the entire ciliated epithelium. In addition, we did not find evidence for tight bacterial adherence. However with our current models, we could not determine whether or not pneumococci accumulate at surface areas where clearance was ineffective due to turbulent fluid flow. Analysis of later processes, in which pneumolysin may be involved, could also not be resolved in more detail due to toxic effects globally affecting the epithelial culture. Both the pathogen specificity of the observed effects and the underlying molecular mechanisms need to be analyzed in further detail.

The observed lesions might be attributable to toxin-mediated, specific targeting of actin or of cellular pathways that regulate the actin cytoskeleton, e.g. via members of the Rho GTPase family such as RhoA, Rac1 and Cdc42 [46]. Well known examples include the glycosyltransferases toxin A and B from *Clostridium difficile* that can inactivate Rho proteins by glycosylation and cause depolymerization of actin fibers, or the fungal cytochalasins that have the ability to bind to actin filaments and to block polymerization and the elongation of actin fibers. When we treated respiratory epithelial cultures with such toxins (data not shown), we noticed uniform reorganization of all F-actin structures (Toxin B), or globular accumulation of actin within the cytoplasm (Cytochalasin D), phenotypes that we never detected upon pneumococcal infections. Pneumolysin has also been reported to cause activation of RhoA and Rac1 GTPases in neuroblastoma cells at sublytic concentrations [16]. However we observed cellular changes of comparable severity when using wildtype or pneumolysin-deficient pneumococci, and no effect was detectable with recombinant pneumolysin alone (2.5 ng/µl; 3 h30 treatment; data not shown), suggesting a pneumolysin-independent mechanism. It also appears possible that pneumococci target two different pathways that separately affect the apical and the junctional F-actin, respectively. In a previous report, treatment of mouse tracheal cell cultures in early epithelial differentiation with RhoA-inactivating *Clostridium botulinum* C3 exotoxin, impaired basal body docking, ciliary axoneme production and apical actin localization, but did not disrupt the epithelial layer, suggesting that the apical membrane organization was selectively altered, whereas the baso-lateral cell junction complexes remained intact [45].

The epithelial barrier is dependent on tight junctions which are connected with the actin cytoskeleton. However, we did not detect abnormal or increased opening of the tight-junctions as a primary defect within the first hours after pneumococcal infection, although the junctional F-actin was clearly affected. It can therefore be speculated that epithelial disorganization occurs prior to separation of the tight junctions, which has been described by others after 48 hours of infection of human respiratory tissue explants [15]. This is further supported by a recent report showing by RT-qPCR of upper respiratory tract lavages in a murine *in vivo* infection model that pneumococci caused downregulation of claudins, key components of tight junctions, via Toll-like receptor-mediated activation of p38 MAPK and TGF-β signaling [47].

The development of a ciliated epithelium involves basal-to-apical polarization, generation of multiple basal bodies from centrioles, their docking to the apical membrane and cilia assembly [48]. In addition, the cells align into a planar structure that lines the tube-like airways, and all cilia are required to be orientated into the posterior-anterior direction, in order to direct mucus flow. The mechanism that controls parts or all of the development and the maintenance of the differentiated morphology is known as the planar cell polarity (PCP) pathway [42], [49]. Accordingly, it has recently been shown that PCP components localize asymmetrically in ciliated respiratory epithelial cells [50] and that PCP signaling is responsible for planar cilia orientation. In addition, PCP defects can cause loss of basal body polarity, disorganization of ciliary beating and impaired directional fluid flow across the ciliated epithelium in *Xenopus* [51]. The apical movement and docking of basal bodies is dependent on the regulated assembly of actin that also involves PCP components [45]. Thus, loss of the columnar shape in a high proportion of cells, distortion of the planar alignment of the epithelial cells and partial detachment of cilia from the apical membrane that we observed, collectively suggest that the maintenance of the differentiated phenotype and possibly the PCP signaling was impaired upon pneumococcal infection.

Another hypothesis predicts a regulated cellular response mechanism rather than a toxin-mediated scenario. Motile cilia might execute sensory functions to determine pathogenic material entrapped in the airway mucus, probably through members of the Toll-like receptors (TLR), a family of pattern recognition receptors [52], [53] located in the ciliary membrane. Further defense mechanisms might then be initiated, such as increased mucus production or immune responses but also epithelial tightening by actin-re-organization at the expense of cilia-mediated epithelial flow. In human patients with mutations in *MyD88* (encoding the intracellular adapter that transduces TLR signals), susceptibility to pneumococcal infections is increased [54]. It can therefore be speculated that impaired TLR-signalling accounts for the inability to properly respond to pneumococcal challenge.

The observation that metabolically active pneumococci were required to attack respiratory epithelial cells in a time-dependent manner suggests that the responsible component is produced and accumulating during progress of the infection. A number of pneumococcal virulence factors have been described including soluble (e.g. pneumolysin) as well as cell-bound (e.g. capsular) components. We did not find evidence for a pneumolysin-dependent mechanism and because inactivated pneumococci also had no detectable effect, the responsible factor(s) was(were) probably not exposed at the bacterial surface under the experimental conditions.

Although both simplified airway infection models have limitations (e.g. lack of distal-to-proximal polarization *in vitro*; the mechanical manipulation during explant preparation; absent mucus in both models), they enable the evaluation of the interaction between two complex components – an intact respiratory epithelium and pneumococci (or other pathogens). It will be important to investigate these processes in the presence of mucus, another major component of the mucociliary clearance mechanism, which protects the epithelium from direct contact with the pathogens, and which adds another step of complexity.

## Supporting Information

Figure S1 **Pneumococcal infection causes time-dependent, progressive lesions in the F-actin cytoskeleton and loss of epithelial integrity in respiratory epithelial cells in vitro.** Respiratory epithelial cultures were infected with pneumococci and various (airway) pathogenic and non-pathogenic bacterial species and analyzed for morphological alterations by confocal microscopy after staining of F-actin (phalloidin, red), cilia (acetylated α-tubulin, green) and nuclei (Hoechst33342, blue). 3D illustrations were rebuilt from z-stack serial images. Scale bars, 10 µm. **(A)** In 3D top views, the F-actin cytoskeleton in ciliated respiratory epithelium has a stable “honeycomb-like” architecture, composed of junctional and apical F-actin. During infection with pneumococci (D39), it is progressively converted into an unstable “net-like” structure. The lesions include distortion of the junctional F-actin and loss of apical F-actin. **(B)** 3D profiles demonstrate irregular distortions of the epithelial surface. **(C)** Orthogonal views illustrate the predominant F-actin at the apical cell cortex in the control, and the progressive mislocalization to the lateral cell borders and the cell bodies upon pneumococcal infection. **(D)** The distortions of the polarized epithelium also affect the alignment of cilia and the basal positioning of nuclei. **(E–I)** No comparable aberrations are obtained with various other bacterial species under similar experimental conditions. Group B streptococci, *E. coli* and *S. aureus* had accelerated growth kinetics compared to pneumococci. Group-B streptococci can cause F-actin defects that include increased opening of the tight junctions, indicated by double-lined cell borders (Figure S2). Respiratory epithelia resist infection with non-pathogenic *E.coli* (laboratory strain HB101; 7 h) and only show non-specific toxic effects due to bacterial overgrowth (27 h), such as increased deposition of F-actin at the basal and lateral cell sides. No specific targeting of F-actin is detected with pathogenic *E.coli* (5 h, MOI limited by dilution of the inoculum during infection), but uncontrolled overgrowth (5 h30) causes toxic effects. Under the experimental conditions used for pneumococcal infections *in vitro*, no effects are obtained with *Haemophilus* influenzae (H.I.) and *Staphyllococcus aureus* (S.a.). **(E)** 3D top views. **(F)** 3D profile views. **(G-I)** 3D orthogonal views.
(TIF)

Figure S2 **The integrity of the tight junctions in respiratory epithelial cells is indicated by the sub-cellular localization of junctional F-actin and/or ZO-1.** Ciliated respiratory epithelium grown *in vitro* was analyzed for localization of F-actin (phalloidin staining, red) and the tight junction protein ZO-1 (anti-zona occludens-1 antibodies, yellow) by confocal imaging. Single color and overlay images are annotated. Cilia (anti-acetyleted α-tubulin, green) and nuclei (Hoechst33342, blue) are shown in overlay images as indicated. Scale bars: 10 µm. **(A)** 2D confocal section at the apical cell side shows the two key elements of the F-actin cytoskeleton (apical F-actin surrounding the ciliary bases; junctional F-actin at the cell borders) and demonstrates co-localization of junctional F-actin and ZO-1 with a cortex-like pattern in each cell. **(B)** 3D profile views illustrate the cortex-like structure comprising F-actin and ZO-1 beneath the ciliated apical cell side. **(C)** In orthogonal views, the apical and junctional F-actin elements can readily be discriminated. **(D)** In 3D top views, frequent opening of the tight junctions, indicated by double-lined cell borders, is detected following either F-actin (left) or ZO-1 (right) staining after 6 h of infection with Group B streptococci.
(TIF)

Figure S3 **Distortion of the planar ciliated respiratory epithelial surface, apical F-actin lesions and cilia undocking after pneumococcal infection.** Respiratory epithelial cultures were analyzed for alterations of the F-actin cytoskeleton (phalloidin staining, red) and the ciliated surface (acetylated α-tubulin, green) after pneumococcal infection (3 h). Scale bars: 10 µm. **(A)** With a planar architecture, most of the ciliated surface and the F-actin cytoskeleton appear in one single confocal 2D section at the apical epithelial side (left panels). 3D illustrations show the “honeycomb-like” structure of the F-actin cytoskeleton, composed of junctional and apical F-actin, beneath the ciliated surface, (right panels). **(B)** After infection with wildtype (not shown) and pneumolysin-deficient pneumococci, plyA(-), a non-planar distortion of the epithelial cell alignment, which coincides with severe reduction of the apical F-actin staining is observed. Extended black regions indicate epithelial areas that are outside of the optical 2D sections (left). In the 3D illustration, the ciliated surface appears almost normal, whereas the F-actin cytoskeleton shows a markedly aberrant “net-like” pattern (right). **(C)** Consecutive optical sections at the apical side (z1–z4; left-to-right = apical-to-basal) of ciliated respiratory epithelial cells demonstrate that each cilium (green) enters the cell body through one individual F-actin “frame”. The row-like alignments of the “frames” reflect the orientation of cilia and provide mechanical stability. **(D)** In cultures infected with pneumococci, the reduction of apical F-actin and the increased diameters of the F-actin frames indicate loss of the mechanical support at the ciliary bases. **(E)** Orthogonal views of the apical cell cortex illustrate the exclusive localization of cilia to the cell surface and the F-actin support to the ciliary bases. **(F)** After pneumococcal infection, partial undocking of cilia occurs, indicated by intracellular cilia staining in cells with reduced apical F-actin.
(TIF)

Figure S4 **Severe F-actin disorganization in the ciliated tracheal epithelium upon pneumococcal infection ex vivo.** The F-actin cytoskeleton of the ciliated epithelium from freshly dissected mouse trachea was analyzed for morphological alterations upon pneumococcal infection *ex vivo*. Confocal microscopy was performed on tissue explants (leaving the epithelium attached to the tracheal cartilage) after fluorescence staining of F-actin (phalloidin, red), cilia (acetylated α-tubulin, green) and nuclei (Hoechst33342, blue). The morphologically discontinuous tracheal epithelium lines the non-flexible and spans the flexible cartilage sections. Consecutive z-images in the apical region of the tracheal epithelium (z1–z5) are shown. 3D assemblies of the F-actin cytoskeleton alone are depicted on the right (top views). Scale bar: 10 µm. **(A)** The ciliated epithelium lining a non-flexible cartilage part consists of columnar shaped epithelial cells that are aligned in a planar epithelial structure with a “honeycomb-like” F-actin cytoskeleton. Nuclei localize to the basal side and are not visible. These structures are also observed *in vitro* cultures of airway epithelium. **(B)** The flexible areas of the trachea are spanned by row-like alignments of ciliated and non-ciliated cells. This pattern indicates the distal-to-proximal polarity of the epithelium, which is absent in air-liquid interface cultures. **(C)** The two key elements of the F-actin cytoskeleton (junctional and apical F-actin) that are readily detectable in air-liquid interface cultures can also be identified in the ciliated epithelium of tracheal explants. Cilia orientation is indicated by the row-like alignment of the actin “frames” that surround the ciliary bases at the planar epithelial surface. **(D)** Pneumococcal infection (3 h) *ex vivo* causes severe lesions in the ciliated tracheal epithelium. The disorganization of the F-actin cytoskeleton that affects both apical and junctional F-actin, coincides with loss of the polarized cell architecture, indicated by the appearance of nuclei at the non-planar distorted epithelial surface.
(TIF)

Video S1 **Irregular surface structure and weakened ciliary beating, but with normal frequency, 4** **h after pneumococcal infection.**
(MOV)

Video S2 **Persistent ciliary beating at normal frequency in respiratory epithelia with severe lesions in the F-actin cytoskeleton after pneumococcal infection.** Morphological analyses (right) of the infected cultures directly after functional evaluation (left).
(MOV)
