# Supplementary figures and images for: Mucociliary Clearance Defects in a Murine In Vitro Model of Pneumococcal Airway Infection
Source: PLoS One. 2013 Mar 19;8(3):e59925. doi: 10.1371/journal.pone.0059925 (PMC3602288; doi:10.1371/journal.pone.0059925)

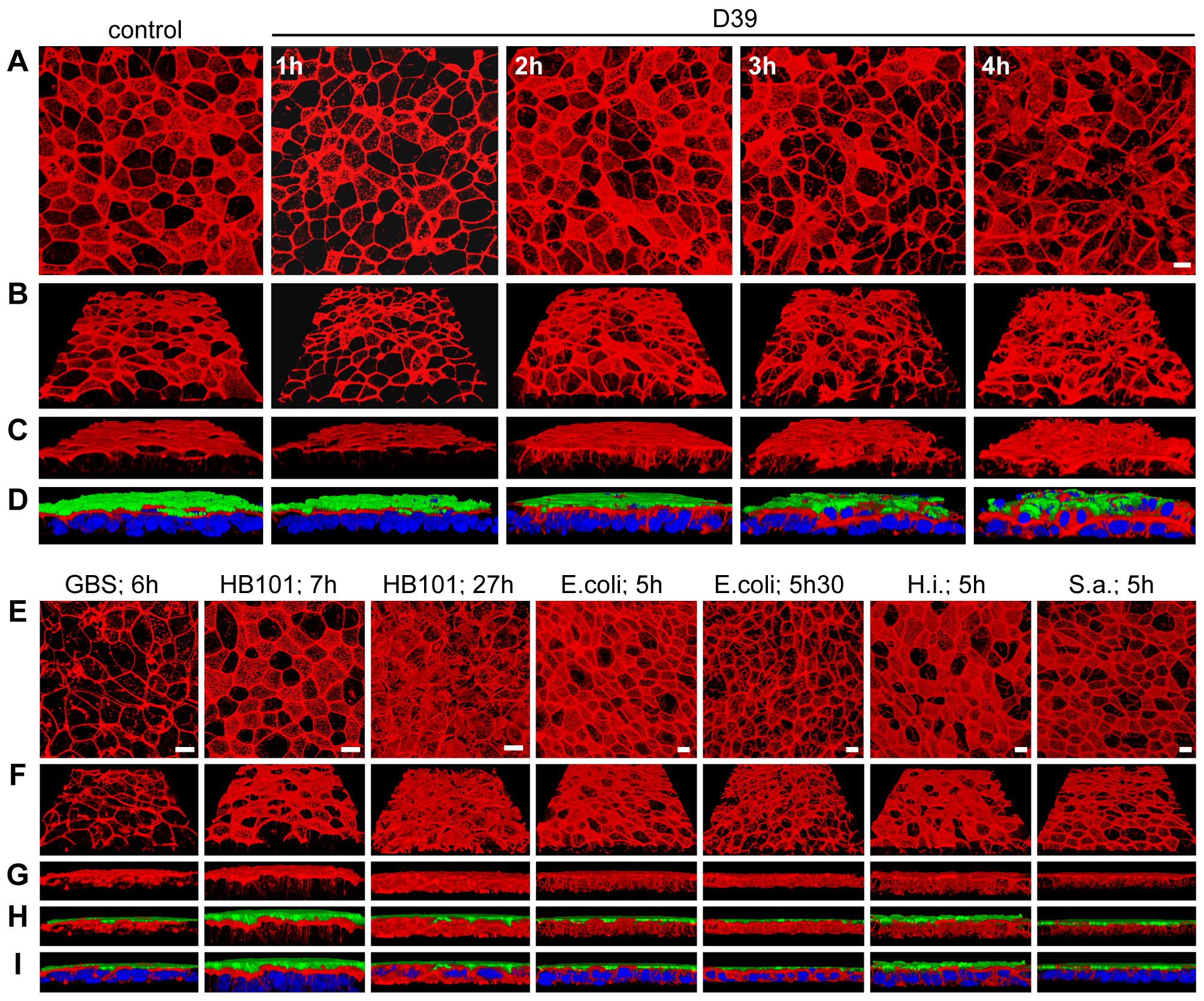

Supplement: Figure S1 — Pneumococcal infection causes time-dependent, progressive lesions in the F-actin cytoskeleton and loss of epithelial integrity in respiratory epithelial cells in vitro. Respiratory epithelial cultures were infected with pneumococci and various (airway) pathogenic and non-pathogenic bacterial species and analyzed for morphological alterations by confocal microscopy after staining of F-actin (phalloidin, red), cilia (acetylated α-tubulin, green) and nuclei (Hoechst33342, blue). 3D illustrations were rebuilt from z-stack serial images. Scale bars, 10 µm. (A) In 3D top views, the F-actin cytoskeleton in ciliated respiratory epithelium has a stable “honeycomb-like” architecture, composed of junctional and apical F-actin. During infection with pneumococci (D39), it is progressively converted into an unstable “net-like” structure. The lesions include distortion of the junctional F-actin and loss of apical F-actin. (B) 3D profiles demonstrate irregular distortions of the epithelial surface. (C) Orthogonal views illustrate the predominant F-actin at the apical cell cortex in the control, and the progressive mislocalization to the lateral cell borders and the cell bodies upon pneumococcal infection. (D) The distortions of the polarized epithelium also affect the alignment of cilia and the basal positioning of nuclei. (E–I) No comparable aberrations are obtained with various other bacterial species under similar experimental conditions. Group B streptococci, E. coli and S. aureus had accelerated growth kinetics compared to pneumococci. Group-B streptococci can cause F-actin defects that include increased opening of the tight junctions, indicated by double-lined cell borders (Figure S2). Respiratory epithelia resist infection with non-pathogenic E.coli (laboratory strain HB101; 7 h) and only show non-specific toxic effects due to bacterial overgrowth (27 h), such as increased deposition of F-actin at the basal and lateral cell sides. No specific targeting of F-actin is detected [file pone.0059925.s001.tif]

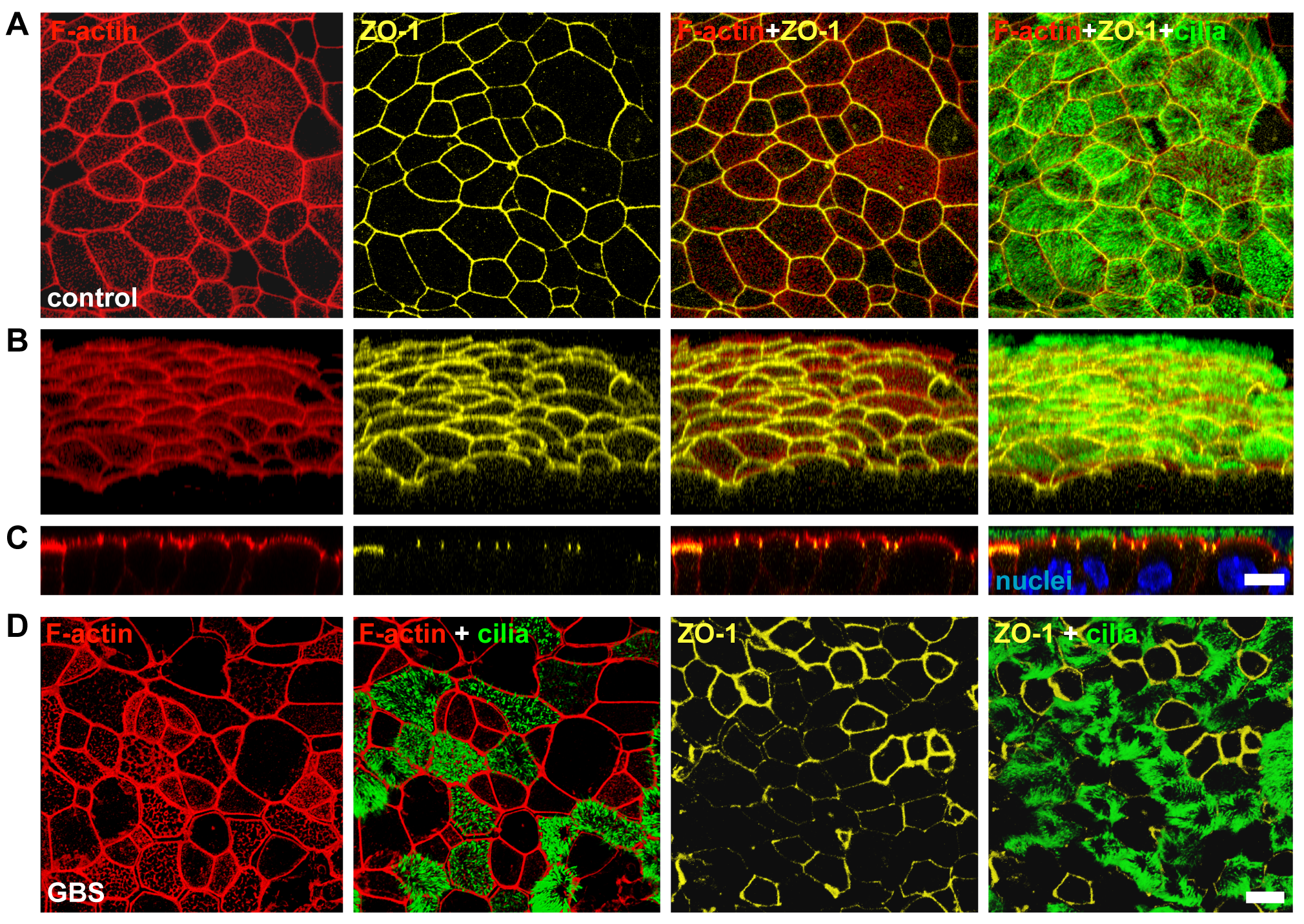

Supplement: Figure S2 — The integrity of the tight junctions in respiratory epithelial cells is indicated by the sub-cellular localization of junctional F-actin and/or ZO-1. Ciliated respiratory epithelium grown in vitro was analyzed for localization of F-actin (phalloidin staining, red) and the tight junction protein ZO-1 (anti-zona occludens-1 antibodies, yellow) by confocal imaging. Single color and overlay images are annotated. Cilia (anti-acetyleted α-tubulin, green) and nuclei (Hoechst33342, blue) are shown in overlay images as indicated. Scale bars: 10 µm. (A) 2D confocal section at the apical cell side shows the two key elements of the F-actin cytoskeleton (apical F-actin surrounding the ciliary bases; junctional F-actin at the cell borders) and demonstrates co-localization of junctional F-actin and ZO-1 with a cortex-like pattern in each cell. (B) 3D profile views illustrate the cortex-like structure comprising F-actin and ZO-1 beneath the ciliated apical cell side. (C) In orthogonal views, the apical and junctional F-actin elements can readily be discriminated. (D) In 3D top views, frequent opening of the tight junctions, indicated by double-lined cell borders, is detected following either F-actin (left) or ZO-1 (right) staining after 6 h of infection with Group B streptococci. (TIF) [file pone.0059925.s002.tif]

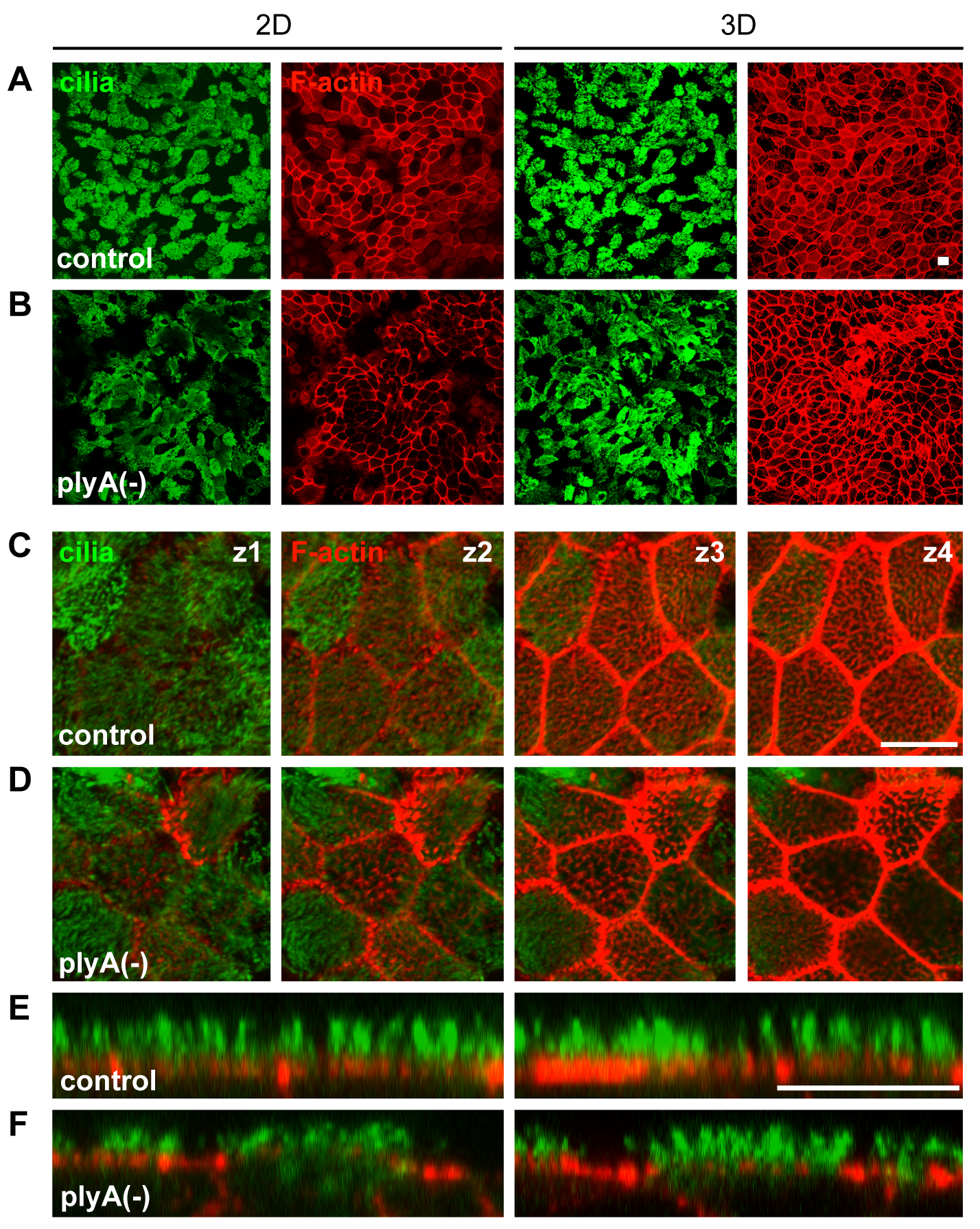

Supplement: Figure S3 — Distortion of the planar ciliated respiratory epithelial surface, apical F-actin lesions and cilia undocking after pneumococcal infection. Respiratory epithelial cultures were analyzed for alterations of the F-actin cytoskeleton (phalloidin staining, red) and the ciliated surface (acetylated α-tubulin, green) after pneumococcal infection (3 h). Scale bars: 10 µm. (A) With a planar architecture, most of the ciliated surface and the F-actin cytoskeleton appear in one single confocal 2D section at the apical epithelial side (left panels). 3D illustrations show the “honeycomb-like” structure of the F-actin cytoskeleton, composed of junctional and apical F-actin, beneath the ciliated surface, (right panels). (B) After infection with wildtype (not shown) and pneumolysin-deficient pneumococci, plyA(-), a non-planar distortion of the epithelial cell alignment, which coincides with severe reduction of the apical F-actin staining is observed. Extended black regions indicate epithelial areas that are outside of the optical 2D sections (left). In the 3D illustration, the ciliated surface appears almost normal, whereas the F-actin cytoskeleton shows a markedly aberrant “net-like” pattern (right). (C) Consecutive optical sections at the apical side (z1–z4; left-to-right = apical-to-basal) of ciliated respiratory epithelial cells demonstrate that each cilium (green) enters the cell body through one individual F-actin “frame”. The row-like alignments of the “frames” reflect the orientation of cilia and provide mechanical stability. (D) In cultures infected with pneumococci, the reduction of apical F-actin and the increased diameters of the F-actin frames indicate loss of the mechanical support at the ciliary bases. (E) Orthogonal views of the apical cell cortex illustrate the exclusive localization of cilia to the cell surface and the F-actin support to the ciliary bases. (F) After pneumococcal infection, partial undocking of cilia occurs, indicated by intracellular cilia staining [file pone.0059925.s003.tif]

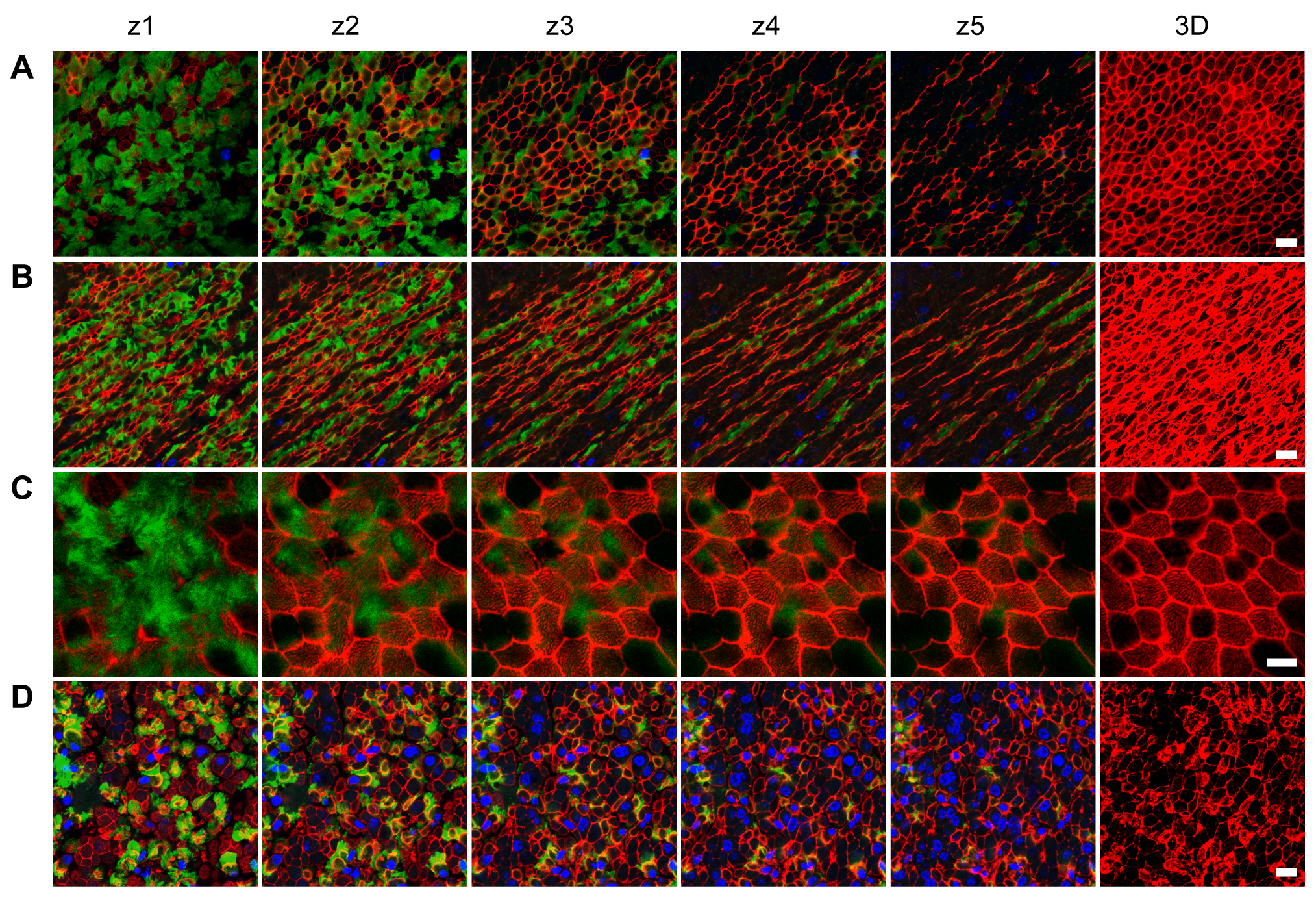

Supplement: Figure S4 — Severe F-actin disorganization in the ciliated tracheal epithelium upon pneumococcal infection ex vivo. The F-actin cytoskeleton of the ciliated epithelium from freshly dissected mouse trachea was analyzed for morphological alterations upon pneumococcal infection ex vivo. Confocal microscopy was performed on tissue explants (leaving the epithelium attached to the tracheal cartilage) after fluorescence staining of F-actin (phalloidin, red), cilia (acetylated α-tubulin, green) and nuclei (Hoechst33342, blue). The morphologically discontinuous tracheal epithelium lines the non-flexible and spans the flexible cartilage sections. Consecutive z-images in the apical region of the tracheal epithelium (z1–z5) are shown. 3D assemblies of the F-actin cytoskeleton alone are depicted on the right (top views). Scale bar: 10 µm. (A) The ciliated epithelium lining a non-flexible cartilage part consists of columnar shaped epithelial cells that are aligned in a planar epithelial structure with a “honeycomb-like” F-actin cytoskeleton. Nuclei localize to the basal side and are not visible. These structures are also observed in vitro cultures of airway epithelium. (B) The flexible areas of the trachea are spanned by row-like alignments of ciliated and non-ciliated cells. This pattern indicates the distal-to-proximal polarity of the epithelium, which is absent in air-liquid interface cultures. (C) The two key elements of the F-actin cytoskeleton (junctional and apical F-actin) that are readily detectable in air-liquid interface cultures can also be identified in the ciliated epithelium of tracheal explants. Cilia orientation is indicated by the row-like alignment of the actin “frames” that surround the ciliary bases at the planar epithelial surface. (D) Pneumococcal infection (3 h) ex vivo causes severe lesions in the ciliated tracheal epithelium. The disorganization of the F-actin cytoskeleton that affects both apical and junctional F-actin, coincides with loss of the polarized cell arc [file pone.0059925.s004.tif]
